# Onion Peel: Turning a Food Waste into a Resource

**DOI:** 10.3390/antiox10020304

**Published:** 2021-02-16

**Authors:** Rita Celano, Teresa Docimo, Anna Lisa Piccinelli, Patrizia Gazzerro, Marina Tucci, Rosa Di Sanzo, Sonia Carabetta, Luca Campone, Mariateresa Russo, Luca Rastrelli

**Affiliations:** 1 Department of Pharmacy, University of Salerno, Via Giovanni Paolo II 132, 84084 Fisciano, Italy; rcelano@unisa.it (R.C.); pgazzerro@unisa.it (P.G.); rastrelli@unisa.it (L.R.); 2 Institute of Bioscience and BioResources, National Research Council, Via Università 100, 80055 Portici, Italy; teresa.docimo@ibbr.cnr.it (T.D.); marina.tucci@ibbr.cnr.it (M.T.); 3 Department of Agriculture Science, Food Chemistry, Safety and Sensoromic Laboratory (FoCuSS Lab), University of Reggio Calabria, Via Salita Melissari, 89124 Reggio Calabria, Italy; rosa.disanzo@unirc.it (R.D.S.); sonia.carabetta@unirc.it (S.C.); 4 Department of Biotechnology and Biosciences, University of Milano-Bicocca, Piazza Della Scienza 2, 20126 Milan, Italy; luca.campone@unimib.it

**Keywords:** sustainable agriculture, onion skin, traditional varieties, flavonols, dietary antioxidants,

## Abstract

Food waste is a serious problem for food processing industries, especially when it represents a loss of a valuable source of nutrients and phytochemicals. Increasing consumer demand for processed food poses the problem of minimizing waste by conversion into useful products. In this regard, onion (*Allium cepa*) waste consisting mainly of onion skin is rich in bioactive phenolic compounds. Here, we characterized the flavonoid profiles and biological activities of onion skin wastes of two traditional varieties with protected geographical indication (PGI), the red “Rossa di Tropea” and the coppery “Ramata di Montoro”, typically cultivated in a niche area in southern Italy. The phytochemical profiles of exhaustive extracts, characterized by ultra-high-performance liquid chromatography coupled with ultraviolet (UV) detection and high-resolution mass spectrometry, revealed that flavonols and anthocyanins were the characteristic metabolite classes of onion skins. Quercetin, quercetin glucosides and their dimer and trimer derivatives, and, among anthocyanins, cyanidin 3-glucoside, were the most abundant bioactive compounds. The potential of onion skins was evaluated by testing several biological activities: ABTS/oxygen radical absorbance capacity (ORAC) and in vitro alpha-glucosidase assays were performed to evaluate the antioxidant and anti-diabetic properties of the extracts and of their main compounds, respectively, and proliferative activity was evaluated by MTT assay on human fibroblasts. In the present study, by observing various biological properties of “Rossa di Tropea” and “Ramata di Montoro” onion-dried skins, we clearly indicated that this agricultural waste can provide bioactive molecules for multiple applications, from industrial to nutraceutical and cosmetical sectors.

## 1. Introduction

The increasing progression of non-communicable diseases (NCD), such as cardiovascular diseases, cancer, and diabetes, as the main causes of deaths worldwide has prompted an improvement of dietary habits [1]. It has been shown that the daily intake of phenolics-rich food or polyphenols-enriched food supplements is highly related to NCD disease prevention. The Mediterranean diet is usually associated with health benefits and favorable life expectancy, due to the daily consumption of different types of vegetables and fruit as a rich source of antioxidants [2,3]. This is particularly true for some key aliments of the Mediterranean diet, such as vegetables belonging to the *Allium* genus, such as onion, garlic, scallion, and chives, whose protective role towards the prevention of several chronic disorders has been widely proved [4]. The multilayer tissue of onion bulbs is a rich source of bioactive compounds belonging to two main chemical groups: the alk(en)yl cysteine sulfoxides and flavonoids. The first group of molecules accounts for the characteristic odor and taste of onion, thus their composition and content determine unique flavors in the different species/varieties [5,6]. Regarding color, different colors of skin and bulbs, ranging from red to yellow, are strictly dependent on flavonoid composition, where the yellow color is mostly due to quercetin and derivatives [7], while red is due to anthocyanins [8]. As recently reported, onion bulbs are ranked among the best sources of dietary flavonoids, mostly belonging to flavonols and anthocyanins [9]. Quercetin glycosides are the predominant flavonols in all onion cultivars whereas the anthocyanins are mainly present in red onions, where they account for approximately 10% of the total flavonoid content of fresh weight [9,10].

Over the past 20 years, world production of onion has raised at least by 25%; [11] reports a production of about 47 million tons every year, becoming the second most important horticultural crop. This is also because lifestyle changes increased the demand for fresh-cut ready-to-use vegetables, including onion. Increased market of processed onion has also led to a higher waste accumulation, and the main European onion producers, Spain, Netherlands, and the UK, reached a waste production of about 50,000 t [12,13,14]. Therefore, to face the high production of food products ingredients and waste, there is the need to find sustainable agricultural processes and develop sustainable solutions for recovering key natural products. The onion skin, being not edible, is fast removed before processing and sale, but is unsuitable as fodder or landfill disposal because of its composition and smell, thus representing the major waste of onion processing. Therefore, sustainable production of *Allium* requires valorizing onion skin by turning this food waste into “food by-product”. By-products from food processing often contain valuable molecules, which can be used as functional ingredients in food, cosmetic, and pharmaceutical industries [15]. In this regard, chemical characterization and knowledge of biological activities of onion skin is necessary for the development of optimal and efficient systems of resource recovery.

The growing demand for food with improved nutritional value and increased sustainability due to the value of its by-products, has increased the interest toward the selection of improved agronomic species and/or local varieties producing food and food ingredients with enriched content of bioactive phytomolecules and improved health beneficial properties [8,10,16,17]. In this regard, the cultivation of superior varieties also offers the opportunity to recover high value by-products. The dried skin of onion is a rich source of flavonols with a high percent of aglycone forms, whereas the content of anthocyanins is limited to red-colored varieties. Most studies have been focused on chemical and biological characterization of edible onion bulbs whereas less attention has been addressed to onion skins because of its inedible nature. [18] reported the characterization of several compounds and of the antioxidant activity of brown and red onion skins from Spanish cultivars, highlighting the brown onion outer layer as the better waste source to valorize. Another study on Californian onions [19], characterized flavonoids in the outer layer of several commercially important onion varieties, highlighting that Californian varieties are richest in flavonoids than some unmentioned Italian varieties. However, literature on onion skins of Italian varieties, is still very scant and especially regarding red skin onion cultivars. Indeed, two commercially important traditional Italian PGI onion varieties, known as “Rossa di Tropea”” and “Ramata di Montoro” are of great interest. The red “Rossa di Tropea”, from Calabria and the coppery “Ramata di Montoro” from Campania local varieties are outstanding food products with several peculiar organoleptic properties conferring distinctive tastes. The Calabrian variety has a typical red-colored envelope and a unique sweetness, whereas the “Ramata di Montoro” has a coppery skin and a delicate and persisting aroma. [8] reported flavonoids and sugar content in the bulb and skin of “Rossa di Tropea” onion, underlying the highest flavonol concentration in this local cultivar than other red Italian onions [6]. Nevertheless, being the high content of anthocyanins concentrated in the inedible outer layer of “Rossa di Tropea” onion, the health promoting potential was not associated with a possible dietary intake. More recently, [10] reported the identification and quantification of polyphenols of “Rossa di Tropea” and “Ramata di Montoro” Italian varieties, showing “Rossa di Tropea” onion bulbs having higher content of bioactive polyphenols, and both exhibiting a strong protective role from oxidative damage thus highlighting a possible synergistic role of the constituents of onion bulbs.

To valorize further the “Rossa di Tropea” and “Ramata di Montoro” onion cultivation, we focused on onion skins to envisage their potential nutraceutical and pharmaceutical use. In this study, we report the chemical characterization and screening of the biological activities of onion skin extracts from these two southern cultivars. We performed a quali-quantitative characterization by ultra-high-performance liquid chromatography (UHPLC) coupled with UV detection and high-resolution mass spectrometry (UHPLC-UV-HRMS) of polyphenols from “Ramata di Montoro” and “Rossa di Tropea” dried skin onions along with an accurate chemical identification of several distinctive compounds by nuclear magnetic resonance (NMR) techniques. Moreover, since phytochemicals can be used in different industrial applications, including the food industry, for the development of functional or enriched foods and food additives, the health industry, for medicines and pharmaceuticals, and/or functional ingredients for cosmetics, we analyzed the biological activities of the onion skin extracts and of their main constituents by evaluation of their antioxidant activities by ABTS and oxygen radical absorbance capacity (ORAC) methods. In addition, we analyzed the potential of the extracts as modulators of postprandial glucose blood levels by testing their activity as alpha-glucosidase inhibitors, and evaluated cell viability by MTT assay on human fibroblasts to assess their safe use in a cosmetic dermatological formulation. by characterizing onion waste, our study aims to promote its use for the production of various crucial bioactive components as step toward sustainable production.

## 2. Materials and Methods

### 2.1. Reagents and Materials

Ultrapure water (18 MΩ) was obtained using a Milli-Q purification system (Millipore, Bedford, TX, USA). MS-grade water (H_2_O), and acetonitrile (CH_3_CN) were supplied by Romil (Cambridge, UK). Analytical grade methanol (MeOH), CH_3_CN, ethyl acetate, n-buthanol (n-BuOH), chloroform, formic acid (HCOOH) and absolute ethanol (EtOH) were supplied by Carlo Erba Reagents (Milan, Italy). Deuterated methanol (99.8%, CD3OD), dimethyl sulfoxide (DMSO), sephadex LH20, MS-grade formic acid (HCOOH), quercetin, isorhamnethin, kaempferol, (±)-6-hydroxy-2,5,7,8-tetramethylchromane-2-carboxylic acid (Trolox) and 2,2′-azinobis (3-ethylben-zothiazoline-6-sulphonic acid) diammonium salt (ABTS), sodium fluorescein and 2,2′-azobis(2-methylpropionamidine) dihydrochloride were provided by Merk Chemical (Milan, Italy). Cyanidine-3-*O*-glucoside (CyG) was purchased from Extrasynthese (Lyon, France).

### 2.2. Plant Material and Exhaustive Extraction

Outer dry protective layers of brown skin onion bulbs (*Allium cepa* L.) of “Ramata di Montoro” were supplied by the company “Gaia Società Semplice Agricola” (Montoro, Avellino, Italy) located in the production area of onion PGI “Ramata di Montoro”. Outer dry protective layers of red skin onion bulbs (*Allium cepa* L.) of PGI “Rossa di Tropea” were provided from Calabrian farmers which organically cultivated and harvested “Rossa di Tropea” onions in Calabria region (Tropea, Vibo Valentia, Italy). Samples were collected after the harvest and naturally dried then before being grounded and sieved according to [16] (Figure S1). The powders were used for exhaustive extraction.

Exhaustive extraction was performed using ultrasound-assisted solid-liquid extraction (UAE) at 25 °C in a thermostat-controlled ultrasound bath (Labsonic LBS2, Treviglio, Italy) at the frequency of 20.0 kHz. Dried *Allium cepa* skins from “Rossa di Tropea” and “Ramata di Montoro” onion varieties were extracted using aqueous EtOH (70% *v*/*v*) and a matrix/solvent ratio of 1:20 (3 × 30 min). At each extraction cycle, after centrifugation 10 min at 9000 g, the extracts of each variety were pooled, filtered (Whatman No. 1 filter) and freeze-dried (freeze dryer Alpha 1–2 LD, Christ, Germany), after the removal of the organic solvent under vacuum at 40 °C in a rotary evaporator (Rotavapor R-200, Buchi Italia s.r.l, Cornaredo, Italy). Extraction yields of 11.6% and 22.6% (g extract per 100 g dry skin, DM) were obtained for “Ramata di Montoro” and “Rossa di Tropea” onion skins, respectively. Exhaustive “Ramata di Montoro” and “Rossa di Tropea” extracts were then used for further chemical characterization and biological tests.

### 2.3. UHPLC-HRMS^n^ Analysis

Identification of phenolic compounds in the exhaustive skin extracts was carried out using a similar approach and same equipment as reported in [16]. The chromatographic analyses were performed using a PLATINblue UHPLC chromatographic system (Knauer GmbH, Berlin, Germany), consisting of two high-pressure pumps, an autosampler, a column temperature control system and a photodiode array detector, coupled to high-resolution mass spectrometer LTQ Orbitrap (ThermoFisher Scientific, Milan, Italy) equipped with an heated electrospray ionization (HESI) source. Chromatographic separation was performed using a Kinetex C18 column (2.1 × 50 mm, 1.7 μm; Phenomenex, Torrance, CA, USA), thermostated at 30 °C, and a binary gradient H_2_O (A) and CH_3_CN (B) both containing 0.1% HCOOH, at a flow of 600 µL / min. The elution gradient used is the following: 2% B, 0–3 min, 2–13% B, 3–5 min, 13% B, 5–9 min, 13–18% B, 9–12 min, 18% B, 12–13min, 18–30% B 13–17 min, 30–50% B, 17–21 min, 50–98% B, 21–22 min, 98% B, 22–27 min. Injection volume was set at 5 μL. The mass spectrometer was used in negative and positive ionization mode, high purity nitrogen (N_2_) was used as sheath gas and auxiliary gas, 30 and 10 arbitrary units, respectively. High purity helium (He) was used as collision gas. Mass spectrometer parameters were as follows: source voltage 4.0 kV; capillary voltage, –33 V; tube lens voltage, –41.4 V; capillary temperature, 300 °C; MS spectra were acquired by full range acquisition covering 140–1500 *m*/*z*. For fragmentation study, a data dependent scan was performed, and the normalized collision energy of the collision induced dissociation (CID) cell was set at 30 eV and the isolation width of precursor ions was set at 2.0 *m*/*z*. The resolution was 60,000 and 7500 for the full mass and the data dependent MS scan, respectively. Phenolic compounds were characterized according to the corresponding mass spectra, accurate mass, characteristic fragmentation, and retention time. Xcalibur software (version 2.2) was used for instrument control, data acquisition, and data analysis.

### 2.4. Isolation and Identification of Onion Skin Flavonols

Major flavonols of onion skin were isolated from “Ramata di Montoro” onion skin using a purification procedure guided by UHPLC-UV analysis (Section 2.5). The exhaustive skin extract was suspended in distilled water and partitioned with ethyl acetate (200 mL × 5) and n-BuOH (150 mL × 3). For each extraction solvent, the organic phases were pooled and vacuum dried with a rotary evaporator at 40 °C (yields of 59% and 15% for ethyl acetate and n-BuOH portions, respectively). Subsequently, a portion of ethyl acetate extract (about 3 g) was fractionated over a Sephadex LH 20 column (60 g, 1 m × 3 cm i.d), using methanol as eluent at a flow of 0.5 mL min^−1^. Fractions (8 mL each) were collected and analyzed by TLC (Si−gel, chloroform/methanol/water, 80: 20: 2, *v*/*v*) and highlighted with Ce (SO_4_)/H_2_SO_4_. Fractions with similar Rf were combined into 12 major fractions (I–XII) and analyzed by UHPLC-UV. The fractions containing target flavonols were further purified by semipreparative high-performance liquid chromatography with refractive index detector (HPLC−IR) on a Luna C18 column (250 × 10 mm i.d., 10 µm, Phenomenex, Bologna, Italy). In detail, fraction IV was separated using MeOH/H_2_O 6:4 *v*/*v* as mobile phase (flow rate of 1.8 mL min^−1^) to yield compound **11** (quercetin 4′ glucoside, QG). Fractions V, X and XII were separated with MeOH/H_2_O 70:30 *v*/*v* (flow rate of 1.8 mL min^−1^) to yield compound **13** (quercetin, Q), **21** (quercetin dimer, Q2) and **22** (quercetin trimer, Q3), respectively. Compounds **19** (Q2Gb) and **18** (Q2Ga) were isolated from fractions VII and VIII using as mobile phase MeOH/H_2_O 65:35 *v*/*v* (flow rate of 2 mL min^−1^). Fraction II contained compounds **2** (Qox) with a degree of purity suitable for NMR analysis. The butanol extract was directly purified by HPLC-IR using MeOH/H_2_O 40:60 *v*/*v* as mobile phase (flow of 3.0 mL min^−1^) to yield compound **6** (quercetin-3, 4′-*O*-diglucoside, QdG). The degree of purity of the isolated compounds was evaluated by HPLC-DAD analysis (220–600 nm). A Bruker DRX 600 spectrometer operating at 599.19 MHz for ^1^H and 150.86 MHz for ^13^C was used for the NMR spectra. For the measurement of the ^1^H NMR and ^13^C NMR spectra in CD_3_OD the relative signal of CHD_2_OD (δH = 3.31, δC = 49.05) was used as standard. NMR data of isolated compounds were compared with literature data [20] to establish their structures.

### 2.5. Quantitative Analysis by UHPLC-UV

Quantitative UHPLC-UV analyses of onion skin extracts were performed using a Dionex Ultimate 3000 UHPLC chromatographic system (ThermoFisher, Milan, Italy), consisting of two ternary pumps, an autosampler, a column temperature control system and a UV/Vis detector. Chromatographic separation was performed using UHPLC-HRMS assay conditions. UV spectra were acquired in the range of 200–600 nm, and three wavelengths, 295, 365, and 520 nm were selected for the detection of target analytes based on their maximum absorbance. External standard method was employed to determine the levels of main skin compounds. Stock solutions of commercial standards (Q and CyG) and isolated standards (Q-dG, QG, Q2 and Q3) were prepared in DMSO at a concentration of 5 mM, and stored at −20 °C. The calibration levels were prepared from the stock solutions by appropriate serial dilutions with MeOH/H_2_0 1:1, *v*/*v*, and analyzed in triplicate. The linearities of the calibration curves were evaluated in the concentration range of 6.25–100 µM for Q, QG and Q-dG, 25–100 µM for Q2Ga, Q2Gb, Q2, Q3, and CyG. The regression curves were tested with the analysis of variance (ANOVA) and linear model was found appropriate over the tested concentration range (R^2^ values > 0.998). The samples were analyzed after appropriate dilutions, to fall within the dynamic calibration range.

### 2.6. Antioxidant Assays

The antioxidant activities (AOAs) of onion skin exhaustive extracts and their major components (Q, QG, Q2, Q3, and CyG) were evaluated using ABTS scavenging capacity and ORAC assays according to [21,22,23] and using the procedures adapted for use in 96-well plates as reported in [24]. A microplate spectrophotometer reader Multiskan Go (Thermo Scientific) and a multimode plate reader EnSpire 2300 (E, PerkinElmer, Waltham, MA, USA) were employed for ABTS and ORAC assays, respectively. ABTS and ORAC assay results were expressed as Trolox equivalent antioxidant capacity (TEAC) per g of extracts (µmol TE/g) or per µmol of pure compound (µmol TE/µmol). In the ABTS assay, the curves of Trolox, pure compounds and onion skin extracts were obtained by plotting concentration (mg mL^−1^ for onion skin extracts and mM for Trolox and standards) against the average % Inhibition of radical absorbances ((Abs_control_ − Abs_sample_)/Abs_control_ × 100). Concentrations corresponding to % Inhibition of 50 (50% I) were extrapolated from curves and TEAC was calculated as:

Trolox concentration_50%I_/Sample concentration_50%I_ × g extract or µM pure compounds.

In ORAC assay, the net area under the FL decay curve (AUC) vs concentration curves were considered, and TEAC values were calculated as:

Trolox concentration_net AUC_/Sample concentration_net AUC_ × g extract or µM pure compounds.

### 2.7. Alpha-Glucosidase Inhibitory Activity

The assay was carried as reported by [25]. 10mg of dried EtOH extract from “Rossa di Tropea” and “Ramata di Montoro” onion skins, were dissolved in EtOH and appropriately diluted in 0.05 M phosphate buffer. A mixture containing 25 μL of extract (final concentrations of 25–150 μg/mL), 25 μL of α-glucosidase (0.2 U ml^−1^, from Baker’s yeast), and 175 μL of phosphate buffer (50 mM, pH 6.8) was left to stand for 10 min at room temperature. The reaction was started by the addition of 25 μL of 23.2 mM *p*-nitrophenyl-α-D-glucopyranoside (2.5 mM) and incubated for 15 min at 37 °C. The assay was conducted in a 96-well plate, and the absorbance was determined at 405 nm using a Bio-Rad microplate reader (Bio-Rad, Richmond, CA, USA). The amount of released *p*-nitrophenol was determined spectrophotometrically, measuring the absorbance of the solution at 405 nm. The percent inhibition was calculated as follows:

Abs 100% − Abs 0%) − (Abs sample − Abs blank)/(Abs 100% − Abs 0%) × 100

where: Abs 100% is the absorbance of 100% enzyme activity (reaction mixture containing only the enzyme and the substrate); Abs 0% is the absorbance of 0% enzyme activity (reaction mixture containing the substrate and without enzyme); Abs sample is the absorbance of the reaction mixture, containing the substrate, enzyme, and the tested extract; Abs blank is the absorbance of the reaction mixture without the enzyme, containing only the substrate and tested extract.

### 2.8. Cell Culture and Cell Viability Assay

Human dermal fibroblast (HDFA) cell line was obtained from Gibco Life Corporation (ThermoFischer Scientific, Milan, Italy) and maintained in M106 medium supplemented with Low Serum Growth Supplement (LSGS), 100 mg/L streptomycin and penicillin 100 IU/mL at 37 °C in a humidified atmosphere of 5% CO_2_. Stock solutions (20 mg/mL) of extracts in DMSO were stored in the dark at 4 °C until use. Appropriate dilutions were prepared in culture medium immediately prior to use. In all experiments, the final concentration of DMSO did not exceed 0.15% (*v*/*v*).

To evaluate cell viability the colorimetric MTT (3-(4,5 di-methylthiazol-2-yl)-2,5-diphenyltetrazolium bromide) metabolic activity assay was performed as described previously. Briefly, 6 × 10^3^ cells/well were seeded in 96-well plates and exposed to increasing concentrations of extracts and purified compounds for 44 h. MTT stock solution (5 mg/mL in PBS, Sigma) was added to each well and incubated for 4 h at 37 °C in humidified 5% CO_2_ atmosphere. The formazan crystals were solubilized with acidic isopropanol (0.1 N HCl in absolute isopropanol). MTT conversion to formazan by metabolically viable cells was monitored by spectrophotometer at an optical density of 595 nm. Each data point represents the average of three separate experiments in triplicate.

### 2.9. Statistical Analysis

Statistical analyses were performed with Sigma Plot The criterion for statistical significance was *p* < 0.05. All values are expressed as means of at least 3 values ± SD.

## 3. Results and Discussion

### 3.1. UHPLC-DAD-HRMS/MS Analysis of “Rossa di Tropea” and “Ramata di Montoro” Onion Skins

**Figure 1:**
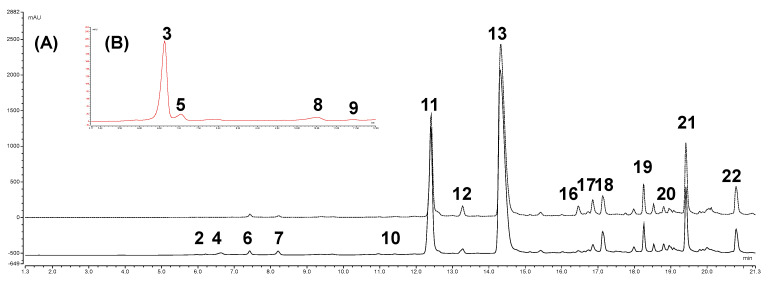
(**A**) UHPLC-UV profile (365 nm) of “Rossa di Tropea” onion skin (solid line) and “Ramata di Montoro” onion skin extract (dashed line); (**B**) UHPLC-UV profile (520 nm) of “Rossa di Tropea” onion skin.

The qualitative profiles of both onion-dried skin extracts were determined by UHPLC-HRMS/MS analysis, taking advantage from the our previous qualitative findings on composition of supercritical fluid extraction (SFE) extract of “Ramata di Montoro” onion skin [16]. In this study, the previous chromatographic conditions were adapted to optimize the detection of anthocyanins in the “Rossa di Tropea” type, particularly 1% formic acid was added to the mobile phases and sample solutions either to improve the chromatographic resolution and to increase the UV absorbance of anthocyanins. Moreover, MS analyses were performed in positive and negative ionization modes to extend the ability of UHPLC-HRMS/MS method to detection and characterization of anthocyanins. Figure 1 shows the UV (365 and 520 nm) profiles of “Ramata di Montoro” and “Rossa di Tropea” onion skin extracts under optimal chromatographic condition. Metabolite assignment were made by comparing retention time, UV/Vis and HRMS/MS data of detected compounds, whenever available, or interpreting MS data combined with chemo-taxonomic data reported in the literature or databases. Twenty-one (**1–22**) major peaks were detected, and their retention times, λ_max_ values and HRMS data are listed in Table 1.

“Ramata di Montoro” and “Rossa di Tropea” onion skin extracts showed a comparable qualitative profile except for the exclusive presence of anthocyanins (**3**, **5**, **8** and **9**) in “Rossa di Tropea”. In both extracts, most of the detected compounds were flavonols, in particular quercetin derivatives, and the isorhamnetin glycosides as minority compounds. No difference in flavonol profiles was observed between the two onion varieties (Figure 1). Both extracts contained flavonol glycosides, with QG (**11**) as major flavonol glycoside. In addition to QG, two mono-*O*-hexoside derivatives (**10** and **11**) and two di-O-hexosides derivatives of quercetin (**4** and **6**) were identified in onion skin extracts (Table 1). Also, isorhamnetin glycosides, namely isorhamnetin-*O*-dihexoside (**7**) and isorhamnetin-*O*-hexoside (**12**), were detected as minority compounds (Table 1). These results are in agreement with data on the flavonols composition of *Allium cepa* [4,8,9,10,17,26]. Besides flavonol glycosides, quercetin oxidation products were also detected in the onion skins. Quercetin is the most abundant flavonol of the onion skin, according to previous studies [9,16,18], and it can undergo oxidation by enzymes contained in plant tissues, such as polyphenol oxidase and peroxidase, generating various oxidation products [27,28]. The oxidized derivatives of quercetin were detected only in the onion skin [16,20,29] and they are considered aging and senescence products [30,31]. It has been hypothesized that they contribute to the browning of the outer onion scales [30,32]. Generally, polyphenol oxidized products in plant foods, are considered unwanted products, as they are responsible for organoleptic changes. However, in some cases (black tea and coffee), the oxidation processes can generate nutritionally and functionally interesting products, worthy of attention and further study [30,31]. In both skin extracts several oxidized derivatives of quercetin were detected: one monomer (**2**), four dimers (**18**, **19**, **20** and **21**) of which three glycosylates and one trimer (**22**) (Table 1). These compounds were tentatively identified by searching the databases for the structure corresponding to the molecular formula calculated from the accurate mass, and comparing their MS/MS spectra with data reported in [16]. In this study, the proposed structures were confirmed by isolation and NMR analysis (Section 3.2). Other compounds found in both onion skins were protocatechuic acid (**1**), isorhamnethin (**17**) and kaempferol (**16**), identified by comparison with reference standards, and two isomers, **14** and **15**, at *m/z* 453.0455 and with calculated molecular formula C_22_H_14_O_11_ (Table 1). According to [20], they might correspond to isomers of quercetin linked to a residue of protocatechuic acid. However, their MS/MS spectra do not correlate with these structures and therefore their identification could not be confirmed.

Anthocyanins are the distinctive feature of red onion varieties and they are responsible for their characteristic red/purple color [9,33]. They are highly concentrated in the skin and outer fleshy layer, whereas in the edible tissue they are limited to a single layer of cells of the epidermal tissue [8]. The most frequently reported anthocyanins in red onion are cyanidin derivatives [9]. Few studies are reported in the literature about the anthocyanins detected in onion skin [8,34,35], but most of the available data refer to the whole onion bulb [9,10]. In “Rossa di Tropea” onion skin extracts, four cyanidin derivatives (**3**, **5**, **8** and **9**) were detected. Compounds **3**, **5**, **8** and **9** showed characteristic UV spectra of anthocyanins with an absorption maximum 520 nm [36]. As is well known, anthocyanins and their derivatives ionized better in the positive mode. Therefore, in HRMS/MS spectra, these compounds were characterized by the product ion at *m*/*z* 287.0550 (ppm 1.02 to 2.52) corresponding to cyanidin aglycone, generated by the loss of one hexoside and one laminaribioside unit for **3** and **5**, respectively, and the malonylhexoside and malonyllaminaribioside unit, for **8** and **9**, respectively. Compound **3** was unequivocally identified as CyG by comparison with reference standards. According to literature data, **5**, **8**, and **9** were tentatively identified as cyanidin 3-laminaribioside, cyanidin 3-malonylglucoside (CymG) and cyanidin 3-malonyllaminaribioside, respectively [34,35]. In general, the obtained results are in agreement with literature data [9]. Cyanidin 3-glucoside and cyanidin 3-laminaribioside are considered the main anthocyanins identified in red onions and the acetylated anthocyanins with malonic acid are the predominant pigments detected in the anthocyanin profile of red onion cultivars [9]. The literature data regarding the phenolic profile of “Rossa di Tropea” red onion are scarce [8,10]. As reported, [8] identified also delphinidin 3-laminaribioside in addition to cyanidin 3-malonylglucoside and cyanidin 3-malonyllaminaribioside in onion skin. In a more recent study conducted on the bulb of the “Rossa di Tropea” red onion, our same anthocyanin profile was reported [10].

### 3.2. Isolation of Major Flavonols of Onion Skin

To confirm the flavonol structures proposed by UHPLC-DAD-HRMS/MS analysis of the “Ramata di Montoro” and “Rossa di Tropea” onion skins (glycosylation site of the glycosidic derivatives and the structure of the oxidation products), and to obtain pure compounds for the quantitative analysis and the evaluation of biological properties, a preparative procedure for isolation of major flavonols was undertaken.

The exhaustive extract of “Ramata di Montoro” onion skin was first fractionated in ethyl acetate and BuOH soluble portions by liquid-liquid partition. Their UHPLC-UV analyses (Figure S2) indicated that ethyl acetate fraction contains all target compounds (**11**, **13**, **18**, **19**, **21** and **22**), except the diglycoside derivatives of quercetin (**6**), present in n-BuOH extract and purified from it by preparative reversed-phase HPLC-IR. The ethyl acetate extract was then purified using gel permeation chromatography followed by reversed-phase HPLC-IR. This procedure (Figure S3) allowed to isolate the compounds QG (**11**), Q (**13**), Q2Ga (**19**), Q2Gb (**18**), Q2 (**21**), Q3 (**22**) and QdG (**6**) from ethyl acetate extract, with a good degree of purity (>90%, UHPLC-DAD). The structures of isolated compounds were elucidated by 1D- and 2D-NMR experiments, confirming the structures hypothesized by the HRMS/MS data (Figure S3).

### 3.3. Quantitative Analysis of “Ramata di Montoro” and “Rossa di Tropea” Onion Skins

The content of major components (QG, Q, Q2Ga, Q2Gb, Q2, Q3, CyG and CymG) of “Ramata di Montoro” and “Rossa di Tropea” onion skins was estimated by UHPLC-UV analysis, to evaluate their abundance and the richness of these by-products. Quantitative determination was performed using calibration curves of corresponding pure compounds, and the data, expressed both as content in onion skin (dry matter) and as concentration of extracts, are listed in Table 2.

In accordance to literature data [10], “Rossa di Tropea” onion skin showed an overall flavonoid content about three times higher than “Ramata di Montoro” variety, 4.7 and 1.5 g/100 g of skin, respectively, also excluding the contribution of anthocyanins (4.4 g/100 g DM) (Table 2). Flavonols were the major bioactive constituents of both studied onion skins, and the estimated amount of “Rossa di Tropea” and “Ramata di Montoro” flavonols fits in the range reported in the literature for skin of different onion varieties, 0.1–4.6 g/100 g DM [18,19]. The most abundant flavonols were QG, representing 34% of the total target compounds in both varieties, and Q (25 and 23% of total in “Rossa di Tropea” and “Ramata di Montoro”, respectively). Our quantitative data agree with the literature data referring to flavonol composition of onion skins [18,19,20,37]. Oxidized derivatives Q2 and Q3 were also relatively abundant (14–17 and 18–22% of total flavonols, respectively). No previous data on their distribution in onion skin are available. Regarding anthocyanins, detected at significant levels only in “Rossa di Tropea” onion skin, CyG resulted the most abundant red pigment and contributed sufficiently to the total flavonoid content (about 8%).

These results highlighted the potential of this food by-product as a rich and economical source of bioactive compounds.

### 3.4. Antioxidant Activity of “Rossa di Tropea” and “Ramata di Montoro” Onion Skin Extracts

Quali-quantitative profiles of “Rossa di Tropea” and “Ramata di Montoro” onion skins highlight that these waste products are an excellent source of Q and its derivatives and of CyG for “Rossa di Tropea” onion skin (Table 2). As is well known, Q represents one of the compounds with the greatest antioxidant activity present in nature [38,39], and the anthocyanins are natural colorant with interesting antioxidant properties [8,40,41,42]. Thus, in this study, the in vitro antioxidant activities of “Rossa di Tropea” and “Ramata di Montoro” onion skin extracts and of their major bioactive compounds were evaluated, to provide a preliminary assessment of the potential of these onion by-products as source of antioxidants and obtain further information regarding the oxidized quercetin derivatives.

When the antioxidant activity is measured by an individual test, the results can reflect only the chemical reactivity under the specific conditions applied for the assay. Therefore, to have a full view on the antioxidant properties of “Rossa di Tropea” and “Ramata di Montoro” onion skins and their bioactive constituents, two different antioxidant assays were used: ABTS (SET/HAT mechanisms) and ORAC (HAT mechanism) assays [43,44,45]. The results expressed in terms of TEAC (µmol TE/g and µmol TE/µmol for extract and pure compounds, respectively) are reported in Table 3. The two onion skin extracts showed similar trends in both assays, with “Rossa di Tropea” exhibiting higher antioxidant properties than “Ramata di Montoro”. Among the tested compounds, Q and CyG resulted the most powerful antioxidants of onion skins, with comparable TEAC values in both assays (Table 3). These data, along with the differences observed in the chemical composition of the onion skins of the two traditional varieties (Table 2), indicate that CyG contributes appreciably to the antioxidant activity of “Rossa di Tropea” extract, as well as the higher content of Q. About the quercetin derivatives, QG and QdG displayed much lower antioxidant activities than their aglycone (Table 3), whereas the oxidated derivatives Q2 and Q3 showed valuable antioxidant properties even if slightly lower than Q. These results are in accordance with previous studies on the flavonoid structure-antioxidant activity relationships [18,19,20,37]. As is common knowledge, the strong antioxidant activity of quercetin depends on the number and position of the hydroxyl groups, especially *o*-dihydroxy (3′-4′), 3- and 5-OH substituents and the 2,3-double bond conjugated with keto function [8,39,46,47]. Therefore, in the case of QG and QdG the glycosylation of hydroxyl groups in position 4′ and/or 3 reduces noticeably their antioxidant properties. Conversely, the oxidative products Q2 and Q3 retain *o*-dihydroxy substituent in the B-ring and consequently the structural criteria underlying the radical scavenging potential. In the same way, the degree and position of hydroxylation, glycosylation and methoxylation in the B-ring of anthocyanins affect their stability and reactivity and thereby antioxidant activity [42]. In the case of cyanidin derivatives, [48] have observed that the glycosylation of cyanidin to cyanidin 3-glucoside increased the antioxidant activity [48]. According to the obtained results we can conclude that both onion skins are a rich source of antioxidants exploitable in different applications as food additives and/or functional ingredients for food supplements, nutraceuticals, and cosmetic products.

### 3.5. Onion Skin Extracts as Source of α-Glucosidase Inhibitors

**Figure 2:**
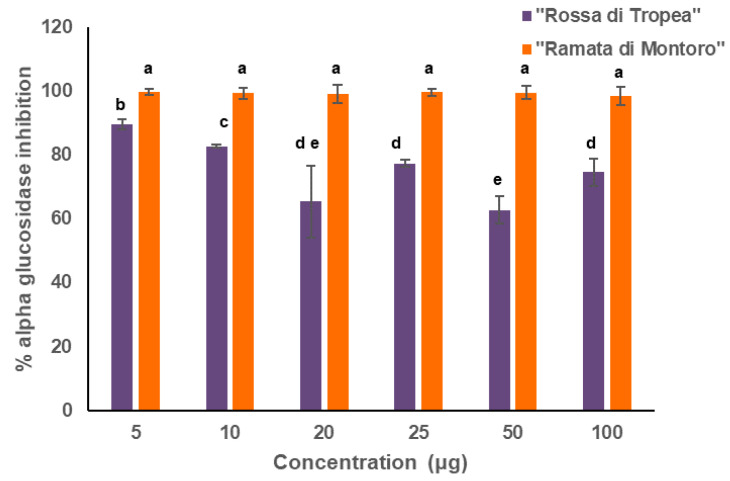
*α*-glucosidase inhibitory activities of “Ramata di Montoro” and “Rossa di Tropea” extracts at different concentrations. Value represents the mean of three replicates. Different letters denote significant differences among tested concentration within each extract by analysis of variance [ANOVA]. Statistical significance was defined as *p* < 0.05, using Tukey’s post hoc test for mean separation.

Several studies report that a strong correlation exists between antioxidant power of plant extracts and α-glucosidase inhibitory activity, and especially onion bulbs have been widely tested for their biological activities including anti-diabetic capabilities [49]. Instead, few studies investigated the anti-diabetic potential of onion skins although they represent the richest source of phenolics. Indeed, [50] reported a higher alpha-glucosidase inhibitory activity of the skin extract than of the onion pulp one for a Korean onion variety, about 90% vs 40%, respectively. Therefore, the potential of “Ramata di Montoro” and “Rossa di Tropea” onion skin extracts for the in vitro α-glucosidase inhibitory activity were assayed toward *Saccharomyces cerevisiae* alpha-glucosidase enzyme. First, to understand whether the extracts could show a different inhibitory capability according to their concentration, a range of concentrations from 5 to 100 μg of extracts were tested (Figure 2). Our data indicated that “Ramata di Montoro” extracts showed an almost 100% capability to inhibit alpha-glucosidase enzyme at each tested concentration, whereas “Rossa di Tropea” extract had the highest inhibitory effect of about 90% only at the lowest tested concentration and its inhibitory power was almost inversely related to the concentration increase.

To estimate the correlation between major flavonoids of “Ramata di Montoro” and “Rossa di Tropea” onion skins and the high α-glucosidase inhibitory activity observed, the α-glucosidase inhibitory activity of the extracts was also compared to pure compounds in a range of concentrations mimicking the actual content when 25 ug of each extract are tested. As shown in Table 4, at each tested concentration CyG, QdG, and QG showed a very low activity, whereas Q, Q2, Q3 showed an almost total enzyme inhibition within the range of tested concentrations. This is not surprising since several studies have reported that quercetin glycosylation strongly reduces its inhibitory effect [51]. Similarly, CyG from “Rossa di Tropea” onion skin extract showed a very low inhibitory potential in agreement with Chen et al. [52], who recently showed that among the major Cinnamon anthocyanins, cyanidin behaves as powerful α-glucosidase inhibitor, while cyanidin 3-rutinoside and cyanidin 3-glucoside did not show inhibitory activity on α-glucosidase. As reported by [53], the increase in hydrophilicity by glycosylation, especially in the 3 position, determines a significant decline of the inhibitory effect, most likely because the free 3-OH is crucial for blocking enzyme active site, as also reported by [54]. Consequently, it is clear that glucosides of cyanidin and quercetin are negatively affecting glucosidase inhibition. Therefore, “Ramata di Montoro” onion skin extract, being devoid of CyG and having also lower content of quercetin glucoside, exhibits a stronger inhibitory activity than “Rossa di Tropea” onion skin extract. In our assay, it can be argued that the use α-glucosidase from yeast *Saccharomyces cerevisiae* could be misleading because the origin of α-glucosidase can influence the activity of the potential inhibitors. Nevertheless, our results agree with data performed on rat intestinal α-glucosidase thus supporting that also in yeast the tested compounds have a constant inhibitory effect. Moreover, α-glucosidase inhibition mechanism of flavonoids and in particular of quercetin, as common plant flavonoid, and main compound in our extracts, has been often investigated [52,55,56,57]. All these studies, reported quercetin acting with as mixed close to non-competitive inhibition type, thus suggesting that this flavonoid can bind both free enzyme and enzyme-substrate complex, whereas 3-OH glycosylated derivatives can vary their inhibitory mechanism toward a competitive inhibition [55,56,57,58]. Overall, our results confirmed that these extracts, act as potent α-glucosidase inhibitors, and suggest that both “Ramata di Montoro” and “Rossa di Tropea” onion skins extracts have the potential to efficaciously contribute as dietary supplements for both the postprandial glycemia control and diabetes-related cellular oxidative stress; nevertheless, further studies will be performed to elucidate the structure-activity relationships of flavonoids inhibitory power in our onion skin extracts.

### 3.6. Onion Skin Extracts Stimulate Cell Viability

Fibroblasts along with keratinocytes, serve to maintain integrity of normal skin. Upon wounding, skin barrier is disrupted, and cells migrate toward the damaged area to regenerate skin by remodeling of the extracellular matrix upon proteolytic degradation and providing novel biosynthesis and deposition of matrix components. To restore cellular integrity is necessary that cells possess high viability and are well proliferating. To test weather onion skin extracts could influence cell proliferation MTT assay was performed.

**Figure 3:**
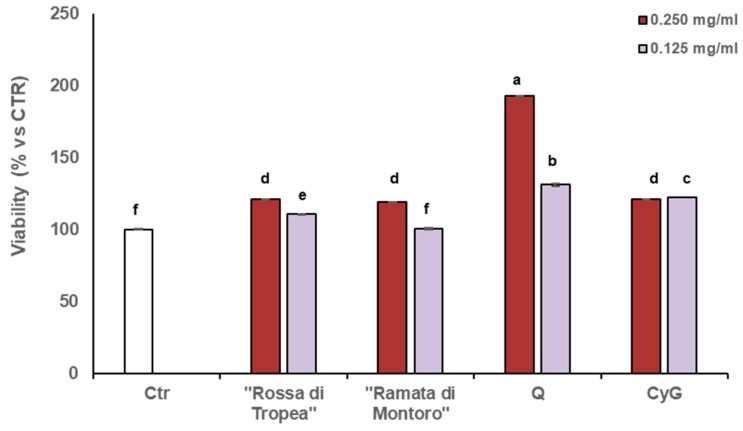
Effects of “Rossa di Tropea” and “Ramata di Montoro” extracts and main compounds on HDFA cell growth. Cells were treated for 48 h with DMSO (Ctrl) or with the indicated concentrations of different extracts. Viability was assessed by MTT assay and was expressed as a percentage of control. Values represent means ± SD. Different letters denote significant differences among tested concentration within each extract by analysis of variance [ANOVA]. Statistical significance was defined as *p* < 0.05, using Tukey’s post hoc test for mean separation.

MTT assay revealed that no cytotoxic effects were detected when cells were cultured with different high concentrations (0.250 mg ml^−1^ and 0.125 mg ml^−1^) of both onion skin extracts. However, in cells cultured with higher concentration of onion waste extracts (0.250 mg ml^−1^) the cell viability was significantly induced after 48 h of culturing, as compared to control untreated cells, being more effective at higher concentration) (Figure 3).

To test the effects of single compounds we selected two of the main constituents, Q and CyG, the latter only present only in “Rossa di Tropea” onion skin extract. Both pure compounds were able to increase cell viability; nevertheless, CyG at both tested concentrations showed a similar effect, not dose-dependent, whereas Q showed the highest proliferative effect to be strictly dose-dependent, namely a higher dose was able to induce a higher proliferative effect. Interestingly, “Rossa di Tropea” extract has comparable content of Q than “Ramata di Montoro”, but the latter is devoid of CyG and derivatives. Taken together our result indicate that both the extracts at higher concentration possess the same proliferative effect but at lower concentration a higher activity can be detected in “Rossa di Tropea” extract, therefore the main effect can be ascribed to Q that at higher extract dilution remains quantitatively high and effective in “Rossa di Tropea” onion skin extract. Absence of cytotoxic effects and indeed the increase in fibroblast proliferation exerted by both onion extracts is a good indication of the potential of the extracts and active ingredients to induce tissue regeneration and facilitate the progression of wound healing.

Our results confirmed the relationship between antioxidant activity due to the presence of bioactive phenolic compounds and cell proliferation, as reported by other studies [59,60]. In particular, this study further highlights that a proactive use of waste to ameliorate regenerative and antioxidant processes can be associated with a wide range of final usage.

## 4. Conclusions

The results presented in this work provide a detailed analysis of skin flavonoid composition of two economically important varieties of *Allium cepa* cultivated in the South of Italy, thus enabling the valorization of value-added products in an otherwise waste product. We are aware that in order to create a sustainable biorefinery approach, mass and energy balances need to be modeled; nevertheless, in this study the use of sun-dried onion matrix, standardized extraction methods with solvent recycling, and low-energy extraction, such as SFE-CO_2_/co-solvent and UAE procedures, ensure the limiting of energetic/environmental burdens. In addition, the screening of biological activities conducted on “Rossa di Tropea” and “Ramata di Montoro” onion skins revealed multiple potential applications of this waste products in several industrial uses as nutraceuticals, food preserving agents, and pharmaceuticals. With a remarkable bioactivity, phytochemicals present in these onion skin extracts can be further investigated for numerous other uses, ranging from sustainable “green” management of agricultural pests to formulation of smart packaging, or formulation of functional food ingredients. Therefore, the multiple re-use of onion waste by-products can be the driving force for the development of sustainable, cost-effective, and efficient technologies for reduction of agricultural and food waste. Hence, our findings can be considered a starting contribution to optimize the sustainable use of this natural resource and make the onion production chain more efficient and sustainable.

## Figures and Tables

**Table 1:** UHPLC-HRMS/MS data of detected compounds in “Ramata di Montoro” (M) and “Rossa di Tropea” (T) onion-dried skin extracts.

N°	Compound	Molecular Formula	RT(_UV_) (min)	Measured (m/z) [M − H]^−^ *(m/z)*	Error (ppm)	Product Ion MS/MS	Measured (m/z) [M + H]^+^ *(m/z)*	Error (ppm)	Product Ion MS/MS	Onion Cultivar
**1**	Protocatechuic acid	C_7_H_6_O_4_	1.0	153.1235	1.6	/	/	/	/	M, T
**2**	2-(3,4-Dihydroxybenzoyl)-2,4,6-trihydroxy-3(2H)-benzofuranone (Qox)	C_15_H_10_O_8_	6.0	317.0303	3.3	299; 191; 207; 273	/	/	/	M, T
**3**	Cyanidin 3-glucoside (CyG)	C_21_H_21_O_11_	6.2	/	/	/	449.1077	−0.3	287	T
**4**	Quercetin dihexoside	C_27_H_30_O_17_	6.6	625.1405	0.9	463; 301	627.1557	0.3	465; 303	M, T
**5**	Cyanidin 3-laminaribioside	C_27_H_31_O_16_	7.0	/	/	/	611.1609	0.3	287	T
**6**	Quercetin 3,4’-diglucoside (QdG)	C_27_H_30_O_17_	7.4	625.1410	−0.3	463; 301	627.1559	0.6	465; 303	M, T
**7**	Isorhamnetin dihexoside	C_28_H_32_O_17_	8.2	639.1571	0.04	477; 315	641.1716	0.5	317; 479	M, T
**8**	Cyanidin 3-malonilglucoside	C_24_H_23_O_14_	10.5	/	/	/	535.1084	0.3	287	T
**9**	Cyanidin 3-malonillaminaribioside	C_30_H_33_O_19_	11.4	/	/	/	697.1609	0.2	287	T
**10**	Quercetin-3-glucoside	C_21_H_20_O_12_	11.6	463.0874	0.6	301	465.1027	−0.2	303	M, T
**11**	Quercetin-4’-glucoside (QG)	C_21_H_20_O_12_	12.4	463.0873	0.8	301	465.1025	−0.6	303	M, T
**12**	Isorhamnetin-O-hexoside	C_22_H_22_O_12_	13.3	477.1031	0.7	315	479.1186	0.5	317	M, T
**13**	Quercetin (Q)	C_15_H_10_O_7_	14.3	301.0350	0.6	179; 151	303.0496	−1.0	285; 257; 229;	M, T
**14**	Protocatecoyl quercetin	C_22_H_14_O_11_	14.8	453.0454	0.4	299	455.0610	0.1	437; 301;	M, T
**15**	Protocatecoyl quercetin	C_22_H_14_O_11_	14.9	453.0455	0.6	299	455.00609	0.1	437; 301;	M, T
**16**	Kaempferol	C_15_H_10_O_6_	16.3	285.0399	2.1	/	287.0548	−0.6	/	M, T
**17**	Isorhamnetin	C_16_H_12_O_7_	16.9	315.0503	1.2	300; 257	317.0656	0.0	302, 285; 257	M, T
**18**	Quercetin dimer 4’-glucoside (Q2Ga)	C_36_H_28_O_19_	17.2	763.1140	−0.2	611; 449;	765.1300	0.3	603; 451	M, T
**19**	Quercetin dimer 4’-glucoside (Q2Gb)	C_36_H_28_O_19_	18.3	763.1139	−0.3	611; 600; 299	765.1298	0.1	603; 585	M, T
**20**	Quercetin dimer hexoside	C_36_H_28_O_19_	18.8	763.1139	−0.2	611; 600; 299	765.1299	0.2	603; 585	M, T
**21**	Quercetin dimer (Q2)	C_30_H_18_O_14_	19.4	601.0617	0.8	449; 299	603.0772	0.5	585; 313; 303	M, T
**22**	Quercetin trimer (Q3)	C_45_H_26_O_21_	20.8	901.0881	−0.2	299; 449; 599; 601	903.1044	0.5	885; 751; 585; 613	M, T

**Table 2:** Flavonoid content levels in “Ramata di Montoro” and “Rossa di Tropea” onion skins and their exhaustive extract.

	“Ramata di Montoro” Onion		“Rossa di Tropea” Onion	
Compounds	Exhaustive Extract (mg/g)	Onion Skin (mg/100 gDM)	Exhaustive Extract (mg/g)	Onion Skin (mg/100 gDM)
CyG	nd	nd	16.2 ± 0.2 ^c^	365.2 ± 4.5 ^c^
CymG ^a^	nd	nd	1.5 ± 0.1 ^a^	34.5 ± 2.3 ^a^
QdG	5.5 ± 0.9 ^a^	64.0 ± 10.5 ^a^	8.8 ± 0.2 ^b^	198.2 ± 4.5 ^b^
QG	44.6 ± 5.2 ^e^	517.7 ± 60.3 ^e^	71.2 ± 3.8 ^g^	1602.9 ± 85.5 ^g^
Q	30.1± 2.5 ^d^	349.5 ± 29.1 ^d^	52.4 ± 2.9 ^f^	1180.0 ± 65.3 ^f^
Q2	22.9 ± 2.8 ^c^	265.5 ± 45.4 ^c^	27.1 ± 4.1 ^d^	608.9 ± 18.8 ^d^
Q2Ga	14.6 ± 2.5 ^b^	168.9 ± 28.9 ^b^	19.4 ± 0.6 ^c^	436.6 ± 13.5 ^c^
Q2Gb	16.4 ± 1.6 ^b^	190.3 ± 18.5 ^b^	17.8 ± 2.6 ^c^	399.6 ± 58.3 ^c^
Q3	29.0 ± 3.7 ^d^	336.7 ± 42.9 ^d^	35.1 ± 9.0 ^e^	790.4 ± 17.3 ^e^

Values are means of three replicates ± SD. nd = not detected. ^a–g^ Expressed as equivalents of CyG. Different superscript letters within each column indicate significant differences among samples (*p* < 0.05, 95% confidence level).

**Table 3:** Antioxidant Activity of “Rossa di Tropea” and “Ramata di Montoro” onion skin extracts and main compounds.

	ORAC	ABTS
	µmol TE/g ± SD	µmol TE/g ± SD
“Rossa di Tropea”	7.82 ± 0.72 ^a^	11.32 ± 1.40 ^a^
“Ramata di Montoro”	4.13 ± 0.29 ^b^	5.77 ± 0.88 ^b^
Compounds	µmol TE/ µmol ± SD	µmol TE/ µmol ± SD
CyG	5.55 ± 1.88	7.21 ± 2.03
QdG	1.01 ± 0.09	2.14 ± 0.55
QG	1.68 ± 1.93	3.60 ± 0.11
Q	5.17 ± 1.76	9.80 ± 0.64
Q2	4.47 ± 3.93	5.58 ± 0.14
Q3	2.42 ± 1.86	6.99 ± 0.15

Values are means of three replicates ± SD. Different superscript letters within each column indicate significant differences among samples (*p* < 0.05, 95% confidence level).

**Table 4:** α-glucosidase inhibitory activity of major compounds of “Ramata di Montoro” and “Rossa di Tropea” onion skin extracts.

Compounds	% Alpha-Glucosidase Inhibition				Concentration Range (C1–C4) *
	C1	C2	C3	C4	
CyG	8.08 ± 1.00	8.58 ± 0.59	7.34 ± 0.41	7.17 ± 0.35	0.15–0.40 μg
QdG	7.30 ± 0.51	6.27 ± 2.03	7.12 ± 0.85	6.98 ± 0.15	0.05–0.25 μg
QG	4.61 ± 0.08	4.55 ± 0.28	5.63 ± 1.22	3.88 ± 0.20	0.75–2.0 μg
Q	99.25 ± 0.32	99.53 ± 0.03	99.46 ± 0.03	99.76 ± 0.43	0.50–1.50 μg
Q2	95.59 ± 1.86	99.07 ± 2.64	97.84 ± 1.80	98.70 ± 2.69	0.15–0.75 μg
Q3	99.27 ± 0.56	99.58 ± 0.07	99.41 ± 0.34	99.45 ± 0.18	0.30–0.90 μg

* C1–C4 are the concentration ranges tested for % alpha-glucosidase inhibitory activity evaluation of the main compounds of “Rossa di Tropea” and “Ramata di Montoro” skin extracts. C1 and C4 correspond to the lowest and highest tested concentrations, respectively. Concentrations have been selected in a range of representative concentrations for each compound when 25 ug of both extracts are tested.

## Data Availability

The authors confirm that the data supporting the findings of this study are available within the article and its Supplementary Materials.

## Supplementary Materials

The following data are available online at https://www.mdpi.com/2076-3921/10/2/304/s1, Figure S1: Picture depicting Rossa di Tropea” and “Ramata di Montoro” varieties and relative onion skins Figure S2: UHPLC-UV profile (365 nm) of ethyl acetate extract (*black*) and n-BuOH extract (*blue*), Figure S3. Preparative procedure for the isolation of the main components of “Ramata di Montoro” exhaustive onion skin extract, Figure S4: Chemical structures of isolated compounds.
