# Supplementary material for: Onion Peel: Turning a Food Waste into a Resource
Source: Antioxidants (Basel). 2021 Feb 16;10(2):304. doi: 10.3390/antiox10020304 (PMC7920456; doi:10.3390/antiox10020304)
Supplement: Supplementary file 1 [file antioxidants-10-00304-s001.pdf]

Supplementary data

Onion Peel: Turning a Food Waste into a Resource

Rita Celano [1,+], Teresa Docimo [2,+], Anna Lisa Piccinelli [1,*], Patrizia Gazzerro [1], Marina Tucci [2], Rosa Di Sanzo [3], Sonia Carabetta [3], Luca Campone [4], Mariateresa Russo [3,*], Luca Rastrelli [1]

[1] Department of Pharmacy, University of Salerno, Via Giovanni Paolo II 132, 84084 Fisciano (SA), Italy; rcelano@unisa.it (R.C), apiccinelli@unisa.it, (A.L.P), pgazzerro@unisa.it, (P.G.), rastrelli@unisa.it (L.R.)

[2] Institute of Bioscience and BioResources, National Research Council,Via Università 100, 80055, Portici (NA), Italy; teresa.docimo@ibbr.cnr.it (T.D), marina.tucci@ibbr.cnr.it (M.T.)

[3] Department of Agriculture Science, Food Chemistry, Safety and Sensoromic Laboratory (FoCuSS Lab), University of Reggio Calabria, Via Salita Melissari, 89124, Reggio Calabria, Italy; mariateresa.russo@unirc.it (M.R.) rosa.disanzo@unirc.it (R.D.S), sonia.carabetta@unirc.it (S.C)

[4] Department of Biotechnology and Biosciences, University of Milano-Bicocca, Piazza Della Scienza 2, I-20126 Milan, Italy; luca.campone@unimib.it

* Correspondence: apiccinelli@unisa.it (A.L.P.), mariateresa.russo@unirc.it (M.T.R.)

\+ These authors contributed equally to this work.

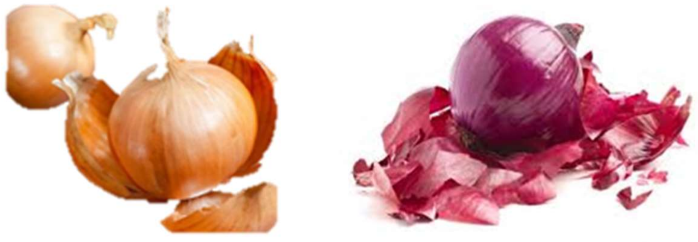

Figure S1. Picture depicting "Rossa di Tropea" and "Ramata di Montoro" varieties and relative onion skins.

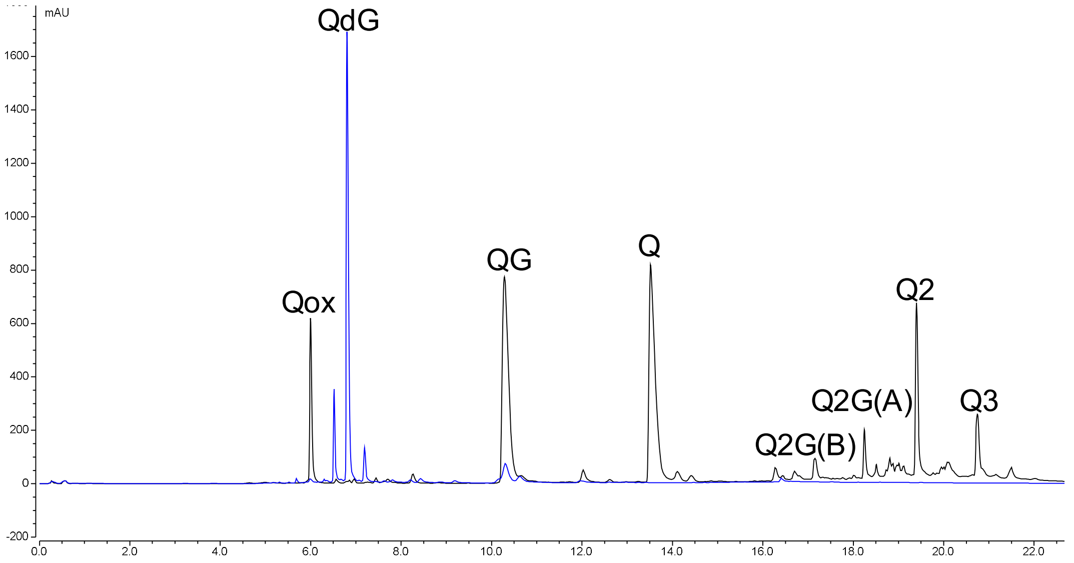

Figure S2. UHPLC-UV profile (365 nm) of ethyl acetate extract (black) and n-BuOH extract (blue)

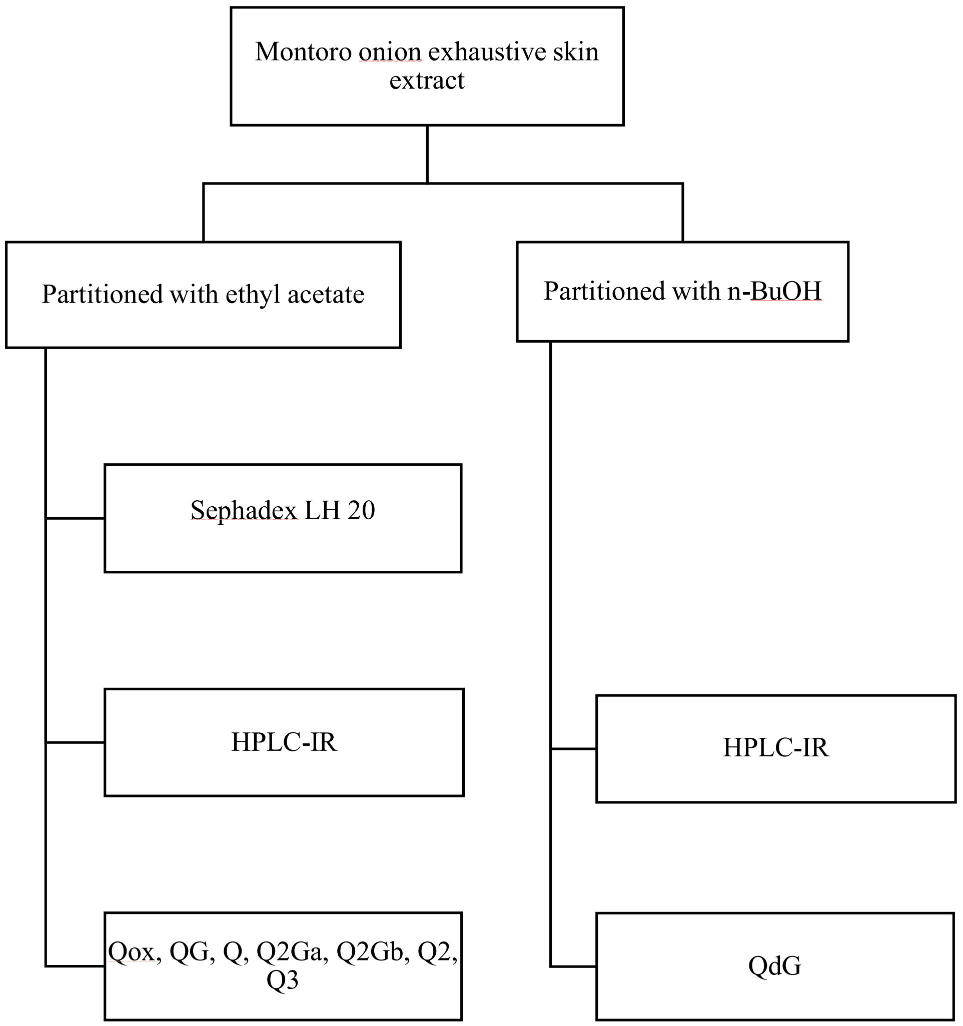

Figure S3. Preparative procedure for the isolation of the main components of Montoro onion exhaustive skin extract

Figure S4 Chemical structures of isolated compounds

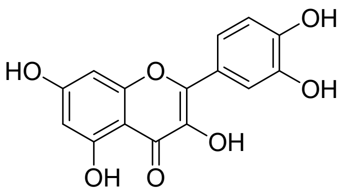

Quercetin (Q)

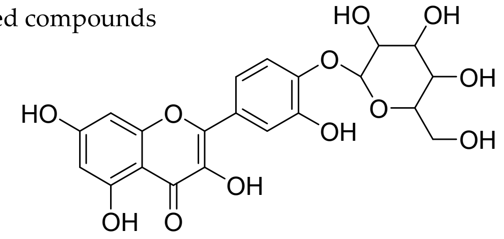

Quercetin 4′ glucoside (QG)

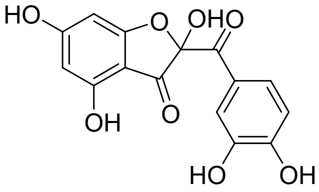

2-(3,4-Dihydroxybenzoyl)-2,4,6-trihydroxy-3(2H)-benzofuranone (Qox)

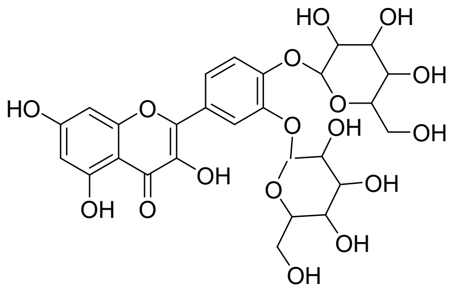

Quercetin 3, 4′ glucoside (Q-dG)

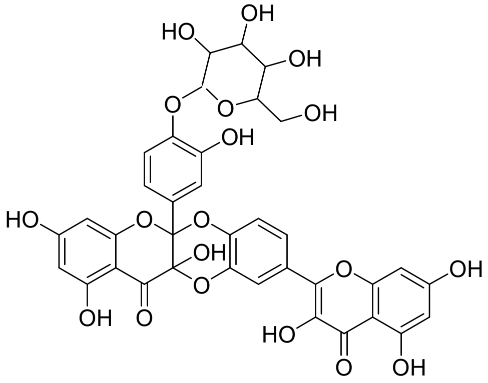

Quercetin dimer 4'-glucoside (Q2G(A))

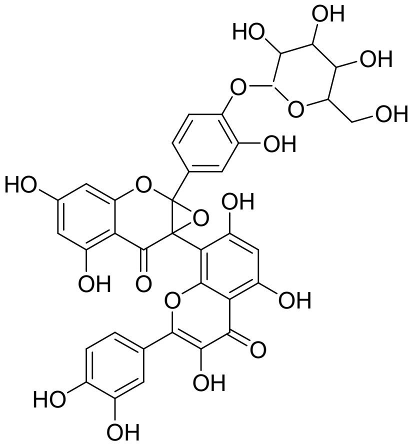

Quercetin dimer 4'-glucoside (Q2G(B))

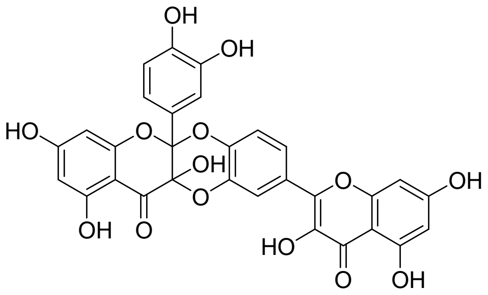

Quercetin dimer

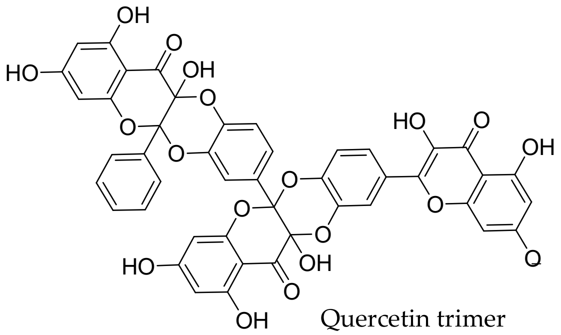

Quercetin trimer
